# Accumulating Comorbidities May Promote Increasing Severity of Obstructive Sleep Apnea with Aging in Males but Not in Females

**DOI:** 10.3390/jpm13010079

**Published:** 2022-12-29

**Authors:** Christopher Seifen, Johannes Pordzik, Katharina Bahr, Lisa Große-Brüggemann, Katharina Ludwig, Berit Hackenberg, Christoph Matthias, Perikles Simon, Haralampos Gouveris

**Affiliations:** 1 Sleep Medicine Center & Department of Otolaryngology, Head and Neck Surgery, University Medical Center Mainz, 55131 Mainz, Germany; 2 Department of Sports Medicine, Disease Prevention and Rehabilitation, Johannes Gutenberg University, 55099 Mainz, Germany

**Keywords:** obstructive sleep apnea, apnea–hypopnea index, apnea index, hypopnea index, age, obstructive sleep apnea and age

## Abstract

Evidence suggests an increasing apnea–hypopnea index (AHI) with aging. However, the effect of aging on sleep-related metrics, especially AHI, has been less frequently investigated within different gender-specific subpopulations by taking prominent confounding factors, e.g., obstructive sleep apnea (OSA)-related comorbidities and body mass index (BMI) into account. Therefore, we retrospectively analyzed 186 first-time polysomnographic (PSG) recordings and medical files of all patients presented to a tertiary university sleep center during a 1-year period. Six groups were formed based on age (over vs. under 55 years) and gender: PSG-related parameters (AHI, apnea-index, and hypopnea-index) were significantly higher in the older mixed-gender cohort (*p* = 0.0001, *p* = 0.0011, and *p* = 0.0015, respectively), and the older female cohort (*p* = 0.0005, *p* = 0.0027, and *p* = 0.001, respectively). Within the older male cohort, the AHI and apnea-index were significantly higher (*p* = 0.0067, and *p* = 0.0135, respectively). Inter-group comparison of the BMI showed no significant difference in any subpopulation. Within the older male cohort there were significantly more patients with arterial hypertension, diabetes mellitus, cardiovascular diseases, and chronic mental health disorders (*p* < 0.0001, *p* = 0.001, *p* = 0.0181, and *p* = 0.0454, respectively). Contrarily, within the female subpopulation there were no significant differences for the aforementioned comorbidities. In conclusion, all investigated sleep PSG-parameters increased among the older subpopulations. We suggest that Osa severity may increase with age due to the increasing accumulation of comorbidities in males, but not in females.

## 1. Introduction

Obstructive sleep apnea (OSA) is the most common type of sleep-disordered breathing. This condition is characterized by recurrent episodes of partial or complete airway obstruction during sleep leading to repetitive apneas or hypopneas. Airway obstruction results from upper airway collapse or anatomic airway obstruction, even though respiratory effort is still present [1]. The clinical signs and symptoms include sleep interruption, snoring, and daytime sleepiness, which can lead to significant impairment in the quality of life. In order to assess the severity of sleep apnea, the apnea–hypopnea index (AHI) is used. The AHI is based on the total number of apneas and hypopneas occurring per hour of sleep; an AHI < 5 is considered normal [2].

OSA represents a major public health issue: In fact, nearly one billion adults aged 30–69 years suffer from OSA worldwide [3]. Population-based epidemiologic studies have uncovered the prevalence of OSA ranging between 4% and 24% in middle-aged people [4]. In addition, the prevalence of sleep apnea has been reported to be 49.7% in men and 23.4% in women aged 40 years or older in a large population-based sample in Western Europe [5]. Further data reported that 88% of men aged 65–69 years had five or more apneic or hypoxic events per hour, increasing to 90% in men aged 70–85 years [6]. The variation in the estimated prevalence is likely to reflect the different health status of the older populations studied and the definition of the disease [7]. For instance, Peppard et al. were among the first authors to show that the apparent increase in AHI with age was strongly correlated with concomitant weight gain [8]. Notably, being overweight, and in particular having an elevated body mass index (BMI) is the strongest risk factor for developing OSA [9]. Conversely, not all obese individuals have OSA. Furthermore, Hoch et al. found that AHI only increased with age among patients with moderate to severe OSA [10]. Contrarily, a recent meta-analysis showed no statistical difference regarding baseline AHI between individuals younger or older than 65 years [11]. In addition, the role of comorbidities in OSA patients has emerged over the years and the association between OSA and medical conditions such as hypertension, diabetes mellitus, cardiovascular disease, pulmonary disease or chronic mental health disorders is a cause for concern, since a bidirectional relationship is suspected [12]. However, it remains controversial whether increasing age alone is associated with an increase in OSA severity, or whether this is due to individual biological changes or the result of more age-related comorbidities.

Therefore, the aim of the present study was to investigate and compare polysomnographic parameters of younger and older individuals in a retrospective analysis of all sleep laboratory polysomnographic recordings of one year, taking baseline study population characteristics, especially OSA-related comorbidities, sex and BMI into consideration.

## 2. Materials and Methods

### 2.1. Study Protocol

The clinical database of our sleep laboratory of a tertiary university medical center was searched retrospectively between 1 January 2020 and 31 December 2020 for all individuals undergoing first-time polysomnography according to the American Academy of Sleep Medicine (AASM) standard guidelines [13]. Each polysomnography was conducted overnight by a licensed technician and interpreted by a sleep specialist. The baseline evaluation of all individuals included assessment of demographic characteristics, e.g., age, sex and body mass index (BMI). In addition, medical records of all individuals were reviewed to search for medical conditions (e.g., arterial hypertension, diabetes mellitus, cardiovascular diseases, pulmonary diseases, and chronic mental health disorders) and daily taken/permanent medication (e.g., antihypertensive drugs, antidiabetic drugs, blood thinners, drugs for the treatment of asthma and chronic obstructive pulmonary disease, and antidepressants). Furthermore, all individuals were reviewed for smoking status (non-smoker, current smoker, ex-smoker, and smoking status not known).

All subjects enrolled in the study received first-time polysomnography in order to screen for OSA based on history of snoring and/or witnessed apneas and/or daytime sleepiness; in other words, OSA screening was due to sleep disorder symptoms and not due to routine health maintenance exam or high risk screening. All individuals aged 18 years and older were included in the study. Individuals under 18 years of age, as well as individuals with positive medical history of heart attack or severe chronic mental health disorders (e.g., schizophrenia, and psychosis) were excluded from the study: It is known, that patients with heart failure are at increased risk for central sleep apnea and the overall prevalence of sleep-disordered breathing among these patients is 40–60% [14]. In addition, patients with severe mental illnesses (e.g., schizophrenia, and psychosis) have increased risk for OSA [15,16]. Moreover, patients with other forms of sleep-disordered breathing than OSA (e.g., central apnea with Cheyne-Stokes respiration or periodic breathing) were excluded from the study.

Six groups were formed: (1) all male and female individuals under 55 years of age were assigned to group “m+f < 55 yrs”; (2) all male and female individuals aged over 55 years were assigned to group “m+f > 55 yrs”; (3) all male individuals under 55 years of age were assigned to group “m < 55 yrs”; (4) all male individuals aged over 55 years were assigned to group “m > 55 yrs”; (5) all female individuals under 55 years of age were assigned to group “f < 55 yrs”; (6) all female individuals aged over 55 years of age were assigned to group “f > 55 yrs”. Based on the median age of the total population, which was 54.2 years, the cut-off point of 55 years was chosen.

For further investigation, the polysomnographic recording of each individual was analyzed for following parameters: AHI, apnea index (AI; total number of apneic events per hour of sleep), hypopnea index (HI, total number of hypoxic events per hour of sleep), snoring index (SI; total number of snoring events per hour of sleep), oxygen desaturation index (ODI, total number of oxygen desaturation events (≥4%) per hour of sleep), mean oxygen saturation (mOS), percentage of oxygen desaturation lower than 90% (t90), total sleep time (TST), percentage of N3 sleep (slow-wave sleep), percentage of REM sleep, and ratio of AHI in the supine position to AHI in the non-supine position (AHI ratio supine/non-supine; a positional OSA when this ratio was ≥2 [17]). 

### 2.2. Ethical Statement

All patients had provided informed consent to the use of their data for research purposes. The data were evaluated in a pseudonymized fashion. Due to this fact and the retrospective nature of the study, the local institutional review board (IRB) was consulted. A separate approval was waived by the local IRB because all retrospective study procedures in this study were in accordance with local data protection and research practices. All procedures were in accordance with the Declaration of Helsinki.

### 2.3. Statistical Analysis

All data were statistically analyzed using JMP 14 (SAS Institute, Cary, NC, USA). Categorical variables were described as number and percentage (%), and continuous variables were described as mean ± standard deviation (SD) for normal distributed or median and interquartile range (IQR) for non-normal distributed values. Within the figures, median and IQR are given. All statistical tests were performed after evaluating normality of distribution with the Shapiro–Wilk test and Kolmogorov–Smirnov test. Comparisons between groups were analyzed using the paired t test, for normal distributed and the Wilcoxon rank-sum test for non-normal distributed values. In order to calculate the statistical significance within the different smoking habits, the Fisher’s test was applied. The results were considered significant when the *p*-value was <0.05. Polysomnographic data were evaluated for statistical power >0.8 at an alpha =0.05 following log-normalization.

## 3. Results

### 3.1. Combined (Male and Female) Study Population

From 1 January 2020 to 31 December 2020 a total of 197 individuals underwent first-time full-night polysomnography in our sleep laboratory. Eleven individuals were excluded from the study due to age under 18 years, medical history of unstable cardiac diseases (e.g., history of heart attack), medical history of severe chronic mental health disorders (e.g., schizophrenia, and psychosis), or forms of sleep-disordered breathing other than OSA (e.g., central apnea). A total of 186 individuals met inclusion criteria and were included in the study.

63 male individuals (63.6%) and 36 female individuals (36.4%) were assigned to group “m+f < 55 yrs”. Age distribution in this group was 22.6–54.9 years (median 44.6 (37.1–51.7) years) and median BMI was 28 (25–32) kg/m². The review of all medical records of group “m+f < 55 yrs” revealed arterial hypertension, diabetes mellitus, cardiovascular diseases, pulmonary diseases and chronic mental health disorders to be present in 27 (27.3%), 3 (3%), 6 (6.1%), 4 (4%), and 11 (11.1%) individuals, respectively. The number of permanent medication taken daily by individuals assigned to group “m+f < 55 yrs” was 1 (0–2). At the time of the medical evaluation, 53 (53.5%) individuals assigned to group “m+f < 55 yrs” were non-smokers, 28 (28.3%) were current smokers, 4 (4%) were ex-smokers and 14 (14.1%) individuals lacked information about their smoking status. A comparison of different smoking statuses did not reveal statistical significance in this group.

Accordingly, 55 male individuals (63.2%) and 32 female individuals (36.8%) were assigned to group “m+f > 55 yrs”. Age distribution in this group was 55.1–85.1 years (median 62.8 (58.6–68.8) years) and median BMI was 30 (26–34) kg/m². The review of all medical records of group “m+f > 55 yrs” revealed arterial hypertension, diabetes mellitus, cardiovascular diseases, pulmonary diseases and chronic mental health disorders to be present in 51 (58.6%), 13 (14.9%), 16 (18.4%), 16 (18.4%), and 18 (20.7%) individuals, respectively. The number of permanent medication taken daily by individuals assigned to group “m+f > 55 yrs” was 3 (2–5). At the time of the medical evaluation, 58 (66.7%) individuals assigned to group “m+f > 55 yrs” were non-smokers, 14 (16.1%) were current smokers, 2 (2.3%) were ex-smokers and 13 (14.9%) individuals lacked information about their smoking status. A comparison of different smoking statuses did not reveal statistical significance in this group. All baseline characteristics of the combined (male and female) study population are shown in Table 1.

### 3.2. Comparison of Sleep Parameters of the Combined (Male and Female) Study Population

**Figure 1:**
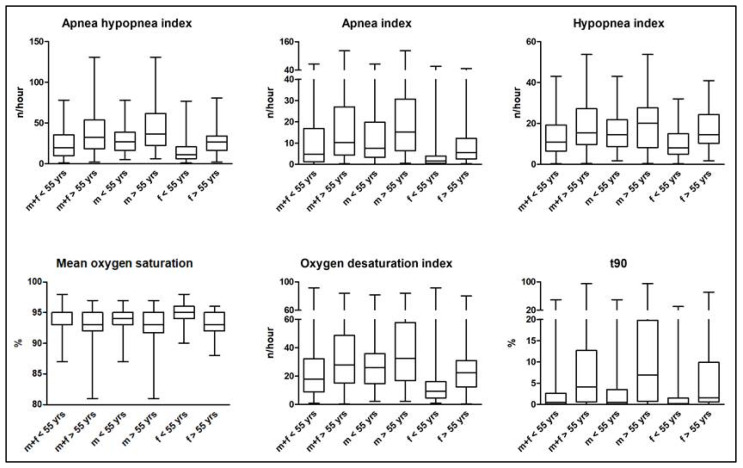
Sleep parameters in the combined (male and female), male and female study population. “m+f < 55 yrs”-all male and female individuals less than 55 years of age; “m+f > 55 yrs”-all male and female individuals aged over 55 years; “m < 55 yrs”-all male individuals less than 55 years of age; “m > 55 yrs”-all male individuals aged over 55 years; “f < 55 yrs”-all female individuals less than 55 years of age; “f > 55 yrs”-all female individuals aged over 55 years. The apnea–hypopnea index (AHI) was significantly higher in the older cohort than in the younger one in the combined (male and female) population, as well as within the subpopulations of men and women (*p* = 0.0001, *p* = 0.0067, and *p* = 0.0005, respectively). Concomitant, AI was significantly higher in all older cohorts (*p* = 0.0011, *p* = 0.0135, and *p* = 0.0027, respectively). Contrarily, HI was higher in all older cohorts, with statistical significance found only in the combined and female subpopulations (*p* = 0.0015, *p* = 0.1044, and *p* = 0.001, respectively). The pulse oximetry measurements of ODI and t90 were found to be significantly higher in all older cohorts (*p* = 0.0015, *p* = 0.0248, and *p* = 0.0031, respectively for ODI; *p* < 0.0001, *p* = 0.0002, and *p* = 0.003, respectively for t90). mOS was found to be significantly lower in all older subpopulations (*p* < 0.0001, *p* = 0.0007, and *p* = 0.0002, respectively). Within the figure, box plots represent median and interquartile range.

The primary goal of this study was to compare sleep parameters in individuals less than 55 years of age (m+f < 55 yrs) to those aged over 55 years (m+f > 55 yrs). In this initial analysis, we compared mixed-gender study population groups. All respiratory parameters, such as AHI, AI, and HI, but not SI, were significantly higher in group “m+f > 55 yrs” (*p* = 0.0001, *p* = 0.0011, *p* = 0.0015, and *p* = 0.0739, respectively). An evaluation of pulse oximetry measurements found ODI and t90 to be significantly higher, and mOS significantly lower in older individuals (*p* = 0.0015, *p* < 0.0001, and *p* < 0.0001, respectively). TST did not differ significantly between groups (*p* = 0.0521). The distribution of sleep stages, more precisely percentages of N3 and REM sleep, did not differ significantly between groups (*p* = 0.0812, and *p* = 0.1677, respectively). Most of the young and aged study population showed supine positional OSA, but no age-related statistical difference was found (*p* = 0.2405). All the above-mentioned sleep parameters of the mixed-gender study population are shown in Table 2 and presented in Figure 1. Given the tested sample size, all significant group differences achieved a statistical power > 0.8 at an alpha = 0.05 following log-normalization of polysomnographic data to account for unequal variance between groups.

### 3.3. Male Study Population

A total of 63 male individuals were assigned to group “m < 55 yrs”. Age distribution in this group was 22.6–54.9 years (median 44.2 (38–51) years) and median BMI was 29 (26–33) kg/m². The review of all medical records of group “m < 55 yrs” revealed arterial hypertension, diabetes mellitus, cardiovascular diseases, pulmonary diseases, and chronic mental health disorders to be present in 16 (25.4%), 1 (1.6%), 3 (4.8%), 3 (4.8%), and 4 (6.3%) individuals, respectively. Regarding pulmonary diseases, bronchial asthma (BA) was found in two cases and chronic obstructive pulmonary disease (COPD) in one case. The number of permanent medication taken daily by individuals assigned to group “m < 55 yrs” was 0 (0–2). At the time of the medical evaluation, 30 (47.6%) individuals assigned to group “m < 55 yrs” were non-smokers, 19 (30.2%) were current smokers, 3 (4.8%) were ex-smokers and 11 (17.5%) individuals lacked information about their smoking status. A comparison of different smoking statuses did not reveal statistical significance in this group.

Accordingly, 55 male individuals were assigned to group “m > 55 yrs”. Age distribution in this group was 55.3–85.1 years (median 61.5 (58.4–68.7) years) and median BMI was 30 (26–34) kg/m². The review of all medical records of group “m > 55 yrs” revealed arterial hypertension, diabetes mellitus, cardiovascular diseases, pulmonary diseases, and chronic mental health disorders to be present in 34 (61.8%), 10 (18.2%), 10 (18.2%), 7 (12.7%), and 10 (18.2%) individuals, respectively. Regarding pulmonary diseases, BA was found in five cases and COPD in two cases. The number of permanent medication taken daily by individuals assigned to group “m > 55 yrs” was 3 (2–5). At the time of the medical evaluation, 36 (65.5%) individuals assigned to group “m > 55 yrs” were non-smokers, 11 (20%) were current smokers, 2 (3.6%) were ex-smokers and 6 (10.9%) individuals lacked information about their smoking status. Comparison of different smoking status did not reveal statistical significance in this group. All baseline characteristics of the male study population are shown in Table 3. 

### 3.4. Comparison of Sleep Parameters of the Male Study Population

After comparing sleep parameters of the combined (male and female) study population, we aimed to compare sleep parameters of only male individuals less than 55 years of age (m < 55 yrs) to those aged over 55 years (m > 55 yrs). In contrast to the combined (male and female) analysis, AHI and AI were the only respiratory parameters being significantly higher in group “m > 55 yrs” compared to group “m < 55 years” (*p* = 0.0067, and *p* = 0.0135, respectively) while HI and SI did not differ significantly between groups (*p* = 0.1044, and 0.3396, respectively). Pulse oximetry measurements of ODI and t90 were found to be significantly higher in older individuals (*p* = 0.0248, and *p* = 0.0002, respectively), while mOS was found to be significantly lower (*p* = 0.0007) in older individuals. Also, TST was significantly lower in older individuals (*p* = 0.0141). The distribution of sleep stages, more precisely percentages of N3 and REM sleep, did not differ significantly between groups (*p* = 0.3459, and *p* = 0.1870, respectively). Supine positional OSA was significantly more prevalent in younger males compared to older individuals (*p* = 0.0048). All the above-mentioned sleep parameters of the male study population are shown in Table 4 and presented in Figure 1. It is noteworthy that, of the polysomnographic data reported as significant for the male collective, only AHI was found to have a statistical power of > 0.8, mainly because of the very high interindividual variance.

### 3.5. Female Study Population

A total of 36 female individuals were assigned to group “f < 55 yrs”. Age distribution in this group was 22.9–54.8 (median 49.7 (36.7–52.8) years and median BMI was 27.5 (23–30) kg/m². The review of all medical records of group “f < 55 yrs” revealed arterial hypertension, diabetes mellitus, cardiovascular diseases, pulmonary diseases and chronic mental health disorders to be present in 11 (30.6%), 2 (5.6%), 3 (8.3%), 1 (2.8%), and 7 (19.4%) individuals, respectively. Regarding pulmonary diseases, BA was reported. The number of permanent medication taken daily by individuals assigned to group “f < 55 yrs” was 1 (0–4). At the time of the medical evaluation, 23 (63.9%) individuals assigned to group “f < 55 yrs” were non-smokers, 9 (25%) were current smokers, 1 (2.8%) were ex-smokers and 3 (8.3%) individuals lacked information about their smoking status. Comparison of different smoking statuses did not reveal statistical significance in this group.

Accordingly, 32 female individuals were assigned to group “f > 55 yrs”. Age distribution in this group was 55.1–83.8 years (median 66.1 (61–70.1) years) and median BMI was 28.5 (24–34.5 kg/m². The review of all medical records of group “f > 55 yrs” revealed arterial hypertension, diabetes mellitus, cardiovascular diseases, pulmonary diseases, and chronic mental health disorders to be present in 17 (53.1%), 3 (9.4%), 6 (18.8%), 9 (28.1%), and 8 (25%) individuals, respectively. Regarding pulmonary diseases, BA was found in six cases and COPD in three cases. In addition, none of the female study participants suffered from anemia in need of specific medical therapy (e.g., iron substitution), the same applies to all male study participants. The number of permanent medication taken daily by individuals assigned to group “f > 55 yrs” was 4 (2–7). At the time of the medical evaluation, 22 (68.8%) individuals assigned to group “f > 55 yrs” were non-smokers, 3 (9.4%) were current smokers, 7 (21.8%) were ex-smokers and zero individuals lacked information about their smoking status. Comparison of different smoking status did not reveal statistical significance in this group. All baseline characteristics of the female study population are shown in Table 5. 

### 3.6. Comparison of Sleep Parameters of the Female Study Population

After comparing sleep parameters of the combined (male and female) and the male study population, we finally aimed to compare sleep parameters of only female individuals less than 55 years of age (f < 55 yrs) to those aged over 55 years (f > 55 yrs). Matching the combined study population, all respiratory parameters, such as AHI, AI, and HI, but not SI, were significantly higher in group “f > 55 yrs”, compared to group “f < 55 yrs” (*p* = 0.0005, *p* = 0.0027, *p* = 0.001, and *p* = 0.0947, respectively). Evaluation of pulse oximetry measurements found ODI and t90 to be significantly higher, and mOS significantly lower in older individuals (*p* = 0.0031, *p* = 0.003, and *p* = 0.0002, respectively). TST did not differ significantly between groups (*p* = 0.8106). The percentage of N3 sleep was significantly higher in younger women (*p* = 0.0224), unlike the percentage of REM sleep, which did not differ significantly between groups (*p* = 0.3846). Most of the young and aged female subgroups showed supine position-related OSA, but no age-related statistical difference was found (*p* = 0.2405). All the above-mentioned sleep parameters of the female study population are shown in Table 6 and presented in Figure 1. In contrast to males, all significant polysomnographic data are reported with a statistical power > 0.8, except for percentage of N3 sleep. 

## 4. Discussion

In this study, we showed that several important sleep parameters differ significantly when younger and older individuals were compared: First, the core parameter for determining the severity of OSA-AHI was significantly higher in older than in younger participants, when considering the combined (male and female) study population, as well as the male and female cohorts. This holds also true for AI, which was significantly higher in all older cohorts. Second, HI was higher in all older cohorts, with statistical significance found only in the combined and female subpopulation. Third, pulse oximetry measurements of ODI and t90 were found to be significantly higher in all older cohorts, while mOS was found to be significantly lower in these subpopulations. Interestingly, SI was not significantly different in any subpopulation analysis.

The aforementioned results are in line with recently published studies: Kopel et al. demonstrated in a large retrospective analysis of 3993 diagnostic sleep test results that AHI less likely is normal as individuals get older [18]. In addition, Ernst et al. proved OSA prevalence to be higher among elderly individuals (>65 years) compared to younger cohorts (18–45 years and 46–65 years) in a retrospective study of 2491 respiratory polygraph recordings [19]. Likewise, Leppänen et al. proved that AHI and duration of apneas, hypopneas, and desaturations increase with increasing age in a retrospective analysis of 1090 individuals with AHI ≥5 [20]. Moreover, Gabbay et al. found that OSA increased with age in both men and women, but men had consistently higher AHIs for each age group [21]. Interestingly, a meta-analysis by Iannella et al. indeed proved a direct correlation between aging and AHI values but found no significant differences in baseline AHI in comparison of individuals younger or older than 65 years [11].

The relationship between OSA severity and age, and also with BMI and medical conditions has recently been of increased interest [9,12]. In fact, the strongest risk factor for developing OSA is being overweight, and in particular an elevated BMI [9]. Obesity increases the risk for OSA by 10–14 times and weight loss reduces the risk for this condition [22]. Notably, neither the combined (male and female) population, nor the male or female subpopulation analyzed in the present study showed significant differences in BMI when younger cohorts were compared with older ones, therefore attenuating a major confounder in the consideration of age-related OSA severity.

Besides BMI, several authors suggested that the age-related increase in OSA severity is due to concomitant age-related increase in OSA-enhancing comorbidities such as chronic heart failure, diabetes mellitus, and renal failure [7,12,23]. This is also the case in the present study: The review of all medical records revealed arterial hypertension, diabetes mellitus, cardiovascular diseases, pulmonary diseases, and chronic mental health disorders to be significantly more prevalent in the combined (male and female) population, as well as the male subpopulation (except for pulmonary diseases). This fact should be taken into account when considering age-related increase in OSA severity.

Interestingly, the data of the present study showed that within the female subpopulation all investigated comorbidities (except for pulmonary diseases) did not differ significantly between the young and old cohort. In summary, within the older cohort of the female subpopulation, all investigated sleep parameters were significantly increased (AHI, AI, HI, ODI, and t90) or decreased (mOS), although there were no significant differences neither in comorbidities nor in BMI compared to the younger cohort. Our results are consistent with a study by Fietze et al. that found a positive linear association between AHI severity and prevalence with age for both men and women. However, OSA onset was later in women [24]. Given that BMI did not differ significantly within all investigated subpopulations, we suggest that OSA severity may increase with age due to the increasing accumulation of comorbidities in males, but not in females.

This finding may be due to the effect of menopause on sleep-disordered breathing in females or may be due to the existing differential systemic pro-inflammatory (or other) effects of intermittent hypoxia between females and males [25,26]. Therefore, these findings provide new evidence suggesting a differential association of sex with the aging process that promotes OSA severity.

Another possible explanation for the increased prevalence of sleep apnea amongst the elderly could be age-related changes in breathing control at sleep onset increasing apneic or hypoxic events during sleep. However, Browne et al. proved stable central breathing control in older people, indicating that aging per se does not promote central sleep apnea [27]. Alternatively, different authors suggested the higher prevalence of OSA in older people may be associated with age-dependent mass reduction or lengthening of pharyngeal skeletal muscles, leading to increase in pharyngeal resistance [28,29]. This thesis might be supported by the fact that the majority of sleep-disordered events are predominantly obstructive.

Due to the cross-sectional study design, the effect of aging on the increase in OSA severity could not be investigated in each individual patient; this limitation may be addressed in future prospective studies. It is acknowledged that future studies are needed to investigate the effects of age on severity of individual AHI and other basic sleep parameters. Notably, the number of permanent medications was significantly higher in all older cohorts, which may also have influenced OSA parameters. Another potential source of error could be that all baseline evaluation of comorbidities as well as the intake of permanent medication was based on self-reporting, although all information was confirmed throughout an in-person interview. Although an attempt was made to compare comorbidities influencing OSA severity or BMI in the different groups, it cannot be excluded that individual diseases or past OSA-specific surgeries were selectively causative for the expression of sleep apnea. Furthermore, it must be acknowledged that all patients examined in our sleep laboratory presented with complaints of clinically relevant sleep apnea syndrome. Thus, our study population does not include asymptomatic OSA patients. Moreover, due to the retrospective study design it was not possible to access the number of women who were menopausal at the time of polysomnography. This limitation makes it impossible to correlate menopause with OSA severity, especially among older women in the study population.

However, to the best of our knowledge, this is the first study to compare different sleep parameters in young versus old mixed-gender cohorts, as well as only male or female subpopulations taking OSA-related baseline characteristics into consideration. This evidence should be considered in the context of an aging global population to prevent the health impacts of OSA and its associated comorbidities, especially in older patients. In addition, it seems that a different strategy of prevention should be followed between females and males.

## 5. Conclusions

All investigated sleep parameters were found to be increased (AHI, AI, HI, ODI, and t90) or decreased (mOS) in the older subpopulations, despite the absence of significant differences in BMI between young and old cohorts. Although comorbidities were more frequently observed among the elderly when mixed-gender cohorts or only males were compared, this was not the case in females. These results provide evidence for an increasing OSA severity with aging in general. Additionally, more specifically, given that sex and BMI did not have an impact on OSA severity in our cohort, we suggest that OSA severity may increase with aging due to the increasing accumulation of comorbidities in males, but not in females. This should be considered in the context of an aging global population to prevent health impact of OSA and its associated comorbidities, especially in older patients.

## Figures and Tables

**Table 1:** Baseline characteristics of the combined (male and female) study population.

	m+f < 55 yrs	m+f > 55 yrs	Between Group Comparison (*p*-Value)
Number of individuals	99	87	
Male participants (%)	63 (63.6)	55 (63.2)	0.9529
Female participants (%)	36 (36.4)	32 (36.8)	0.9529
Age, in years (IQR)	44.6 (37.1–51.7)	62.8 (58.6–68.8)	<0.0001
BMI in kg/m² (IQR)	28 (25–32)	30 (26–34)	0.2874
Arterial hypertension (*n* (%))	27 (27.3)	51 (58.6)	<0.0001
Diabetes mellitus (*n* (%))	3 (3)	13 (14.9)	0.003
Cardiovascular disease (*n* (%))	6 (6.1)	16 (18.3)	0.009
Pulmonary disease (*n* (%))	4 (4)	16 (18.4)	0.0012
Chronic mental health disorder (*n* (%))	11 (11.1)	18 (20.7)	0.072
Number of permanent medication (IQR)	1 (0–2)	3 (2–5)	<0.0001
Number of non-smokers (%)	53 (53.5)	58 (66.7)	0.1774
Number of current smokers (%)	28 (28.3)	14 (16.1)	
Number of ex-smokers (%)	4 (4)	2 (2.3)	
Smoking status not known (*n* (%))	14 (14.1)	13 (14.9)	

“m+f < 55 yrs”-all male and female individuals less than 55 years of age; “m+f > 55 yrs”-all male and female individuals aged over 55 years; BMI-body mass index; IQR-interquartile range.

**Table 2:** Sleep parameters of the combined (male and female) study population.

	m+f < 55 yrs	m+f > 55 yrs	Between Group Comparison (*p*-Value)
AHI (*n*/hour)	19.6 (9.9–35.4)	32.6 (18.3–53.9)	0.0001
AI (*n*/hour)	4.8 (1.2–16.8)	10.2 (4.4–27)	0.0011
HI (*n*/hour)	10.9 (6.4–19.2)	15.4 (9.6–27.2)	0.0015
SI (*n*/hour)	131.4 (34.2–310.7)	194.6 (60.8–407.2)	0.0738
ODI (*n*/hour)	17.9 (8.8–32.2)	27.9 (15.1–48.7)	0.0015
mOS (%)	95 (93–95)	93 (92–95)	<0.0001
t90 (%)	0.5 (0.1–2.6)	4.1 (0.6–12.7)	<0.0001
TST (min)	373.1 (343.3–400.7)	357.6 (304.9–398.9)	0.0521
N3 sleep (%)	14.9 (9.1–22.9)	13.1 (7.7–19.3)	0.0812
REM sleep (%)	17.9 (12.9–22.5)	16.3 (8.7–21.)	0.1677
Ratio supine AHI/non-supine AHI ≥2 (*n* (%))	72 (72.8)	55 (63.2)	0.2405

“m+f < 55 yrs”-all male and female individuals less than 55 years of age; “m+f > 55 yrs”-all male and female individuals aged over 55 years; AHI-apnea–hypopnea index; ratio supine AHI/non-supine AHI ≥2-number (percentage) of individuals with ratio of AHI in supine position to AHI in non-supine position ≥2; AI-apnea index; HI-hypopnea index; ODI-oxygen desaturation index; mOS-mean oxygen saturation; REM-rapid eye movement; t90-percentage of oxygen desaturation lower than 90%; SI-snoring index; TST-total sleep time.

**Table 3:** Baseline characteristics of the male study population.

	m < 55 yrs	m > 55 yrs	Between Group Comparison (*p*-Value)
Number of individuals	63	55	
Age, in years (IQR)	44.2 (38–51)	61.5 (58.4–68.7)	<0.0001
BMI in kg/m² (IQR))	29 (26–33)	30 (26–34)	0.4687
Arterial hypertension (*n* (%))	16 (25.4)	34 (61.8)	<0.0001
Diabetes mellitus (*n* (%))	1 (1.6)	10 (18.2)	0.001
Cardiovascular disease (*n* (%))	3 (4.8)	10 (18.2)	0.0181
Pulmonary disease (*n* (%))	3 (4.8)	7 (12.7)	0.1184
Chronic mental health disorder (*n* (%))	4 (6.3)	10 (18.2)	0.0454
Number of permanent medication (IQR)	0 (0–2)	3 (2–5)	<0.0001
Number of non-smokers (%)	30 (47.6)	36 (65.5)	0.2774
Number of current smokers (%)	19 (30.2)	11 (20)	
Number of ex-smokers (%)	3 (4.8)	2 (3.6)	
Smoking status not known (*n* (%))	11 (17.5)	6 (10.9)	

“m < 55 yrs”-all male individuals less than 55 years of age; “m > 55 yrs”-all male individuals aged over 55 years; BMI-body mass index; IQR-interquartile range.

**Table 4:** Sleep parameters of the male study population.

	m < 55 yrs	m > 55 yrs	Between Group Comparison (*p*-Value)
AHI (*n*/hour)	26.9 (16.3–38.7)	36.5 (22.3–61.7)	0.0067
AI (*n*/hour)	7.5 (3.3–19.8)	15.2 (6.4–30.7)	0.0135
HI (*n*/hour)	14.5 (8.7–21.9)	20.1 (8.2–27.6)	0.1044
SI (*n*/hour)	142.7 (48.5–406.9)	282.7 (49.4–414)	0.3396
ODI (*n*/hour)	26 (14.6–35.9)	32.5 (16.8–57.8)	0.0248
mOS (%)	94 (93–95)	93 (91.8–95)	0.0007
t90 (%)	0.5 (0.1–3.5)	6.9 (0.7–19.8)	0.0002
TST (min)	386.8 (351.1–413.8)	353.3 (297.8–398.9)	0.0141
N3 sleep (%)	14 (8.4–19.1)	12.7 (8–20.1)	0.3459
REM sleep (%)	16.7 (12.9–23)	16.5 (9.7–30.3)	0.1870
Ratio supine AHI/non-supine AHI ≥2 (*n* (%))	52 (82.5)	32 (58.2)	0.0048

“m < 55 yrs”-all male individuals less than 55 years of age; “m > 55 yrs”-all male individuals aged over 55 years; AHI-apnea–hypopnea index; AI-apnea index; ratio supine AHI/non-supine AHI ≥2-number (percentage) of individuals with ratio of AHI in supine position to AHI in non-supine position ≥2; HI-hypopnea index; ODI-oxygen desaturation index; mOS-mean oxygen saturation; REM-rapid eye movement; t90-percentage of oxygen desaturation lower than 90%; SI-snoring index; TST-total sleep time.

**Table 5:** Baseline characteristics of the female study population.

	f < 55 yrs	f > 55 yrs	Between Group Comparison (*p*-Value)
Number of individuals	36	32	
Age, in years (IQR)	49.7 (36.7–52.8)	66.1 (61–70.1)	<0.0001
BMI in kg/m² (IQR)	27.5 (23–30)	28.5 (24–34.5)	0.4713
Arterial hypertension (*n* (%))	11 (30.6)	17 (53.1)	0.0582
Diabetes mellitus (*n* (%))	2 (5.6)	3 (9.4)	0.5468
Cardiovascular disease (*n* (%))	3 (8.3)	6 (18.8)	0.2036
Pulmonary disease (*n* (%))	1 (2.8)	9 (28.1)	0.0019
Chronic mental health disorder (*n* (%))	7 (19.4)	8 (25)	0.5816
Number of permanent medication (IQR)	1 (0–4)	4 (2–7)	0.0011
Number of non-smokers (%)	23 (63.9)	22 (68.8)	0.1137
Number of current smokers (%)	9 (25)	3 (9.4)	
Number of ex-smokers (%)	1 (2.8)	7 (21.8)	
Smoking status not known (*n* (%))	3 (8.3)	0 (0)	

“f < 55 yrs”-all female individuals less than 55 years of age; “f > 55 yrs”-all female individuals aged over 55 years; BMI-body mass index; IQR-interquartile range.

**Table 6:** Sleep parameters of the female study population.

	f < 55 yrs	f > 55 yrs	Between Group Comparison (*p*-Value)
AHI (*n*/hour)	11.1 (6.1–20.8)	26.6 (16.4–34)	0.0005
AI (*n*/hour)	1.55 (0.5–3.9)	5.6 (2.5–12.2)	0.0027
HI (*n*/hour)	8.1 (4.9–15)	14.5 (10.2–24.4)	0.001
SI (*n*/hour)	90.8 (25–207.5)	141.5 (65.4–310.5)	0.0947
ODI (*n*/hour)	9.3 (4.6–16.1)	22.4 (12.4–31)	0.0031
mOS (%)	95 (94–96)	93 (92–95)	0.0002
t90 (%)	0.2 (0–1.5)	1.6 (0.6–9.9)	0.003
TST (min)	360.5 (334.7–384.8)	361.2 (318–398.7)	0.8106
N3 sleep (%)	20.6 (11.9–25)	13.6 (7.14–19.2)	0.0224
REM sleep (%)	18.6 (13.3–21.3)	13.8 (7.9–21.9)	0.3846
Ratio supine AHI/non-supine AHI ≥2 (*n* (%))	20 (55.6)	23 (71.9)	0.0658

“f < 55 yrs”-all female individuals less than 55 years of age; “f > 55 yrs”-all female individuals aged over 55 years; AHI-apnea–hypopnea index; ratio supine AHI/non-supine AHI ≥2-number (percentage) of individuals with ratio of AHI in supine position to AHI in non-supine position ≥2; AI-apnea index; HI-hypopnea index; ODI-oxygen desaturation index; mOS-mean oxygen saturation; REM-rapid eye movement; t90-percentage of oxygen desaturation lower than 90%; SI-snoring index; TST-total sleep time.

## Data Availability

The data presented and analyzed in this study are available on reasonable request by the corresponding author.
